# M1 Polarization but Anti-LPS-Induced Inflammation and Anti-MCF-7 Breast Cancer Cell Growth Effects of Five Selected Polysaccharides

**DOI:** 10.1155/2020/9450246

**Published:** 2020-03-25

**Authors:** Hsiao-Chien Lin, Jin-Yuarn Lin

**Affiliations:** Department of Food Science and Biotechnology, National Chung Hsing University, Taichung 40227, Taiwan

## Abstract

Five potential polysaccharides from guava seed (GSPS), common buckwheat (CBPS), bitter buckwheat (BBPS), red Formosa lambsquarters (RFLPS), and yellow Formosa lambsquarters (YFLPS) were selected to measure their effects on mouse peritoneal macrophages in the absence or presence of lipopolysaccharide (LPS). Macrophage-conditioned media (MCM) in the absence or presence of 5 selected polysaccharides were prepared to treat MCF-7 cells. The cell viability was determined using 3-(4,5-dimethylthiazol-2-diphenyl)-2,5-tetrazolium bromide (MTT) assay. Proinflammatory (also known as M1 type) (interleukin- (IL-) 1*β*, IL-6 and tumor necrosis factor- (TNF-) *α*) and anti-inflammatory (also known as M2 type) (IL-10) cytokines secreted by macrophages were determined using ELISA. The relationship between MCF-7 cell growth and M1/M2 cytokine secretion profiles in the corresponding MCM were delineated. The results showed that 5 selected polysaccharides, except BBPS, significantly (*P* < 0.05) and dose-dependently increased M1 (IL-1*β* + IL-6 + TNF-*α*)/M2 (IL-10) cytokine secretion ratios by macrophages in the absence of LPS, suggesting that four selected polysaccharides have M1 polarization property. However, all of 5 selected polysaccharides significantly (*P* < 0.05) decreased proinflammatory (IL-1*β* + IL-6 + TNF-*α*)/anti-inflammatory (IL-10) cytokine secretion ratios by LPS-stimulated macrophages, exhibiting that all of the 5 selected polysaccharides, particularly GSPS, have anti-inflammatory potential. All MCM prepared with these selected polysaccharides (except YFLPS) significantly enhanced their inhibitory effects on MCF-7 cell growth. A negative correlation was noted between MCF-7 cell viabilities and M1/M2 cytokine secretion ratios ((IL-6 + TNF-*α*)/IL-10) in the corresponding MCM, suggesting that increases in M1 macrophages in the tumor microenvironment might inhibit MCF-7 cell growth. Particular polysaccharides including RFLPS, GSPS, YFLPS, and CBPS may increase the percentage of M1 macrophages in the tumor environment and further inhibit MCF-7 cell growth via immunotherapy.

## 1. Introduction

Macrophages that are inflammatory cells can be activated during inflammation process resulting from bacteria or endotoxin invasion to combat pathogens, thereby reducing the damage to the body [1]. Activated macrophages produce proinflammatory cytokines (also known as M1-type cytokines), such as interleukin- (IL-) 1*β*, IL-6 and tumor necrosis factor- (TNF-) *α*, to recruit more white blood cells to the inflammation site and enhance the inflammation status [2]. Mild or acute controlled inflammation is a normal and essential process for protection; however, chronic or repeatedly uncontrolled inflammation might cause inflammation-related diseases such as cancers. Beyond enhancing inflammation and activating the immune system, macrophages via cytokines release are also found to play an important anti-inflammatory role. A balanced mechanism to regulate excess inflammation exists in the body via producing anti-inflammatory cytokines, such as IL-10 [3]. Consequently, the secretion profile of proinflammatory (M1) and anti-inflammatory (M2) cytokines by macrophages may reflect the inflammation status *in vitro* or *in vivo* [4, 5]. Active phytochemicals with anti-inflammatory potential such as polysaccharides have been proven anti-inflammatory effects through regulating pro- and anti-inflammatory cytokine profiles [6].

The cell differentiation of macrophages has been related to the development of chronic diseases such as cancers. Macrophages can be classified into two main groups designated as M1 and M2, although this dichotomy has been challenged and further complexity has been reported. M1-type macrophages encourage inflammation that help to clear infection and may inhibit tumor cell growth, whereas M2-type macrophages decrease inflammation through producing an anti-inflammatory cytokine IL-10 and improve tissue repair [7]. Macrophages may infiltrate a number of tumors; however, tumor-associated macrophages (TAMs) are mainly M2-type macrophages that seem to actively promote tumor growth and progression [8]. In contrast, M1 phenotype macrophages in the tumor environment may help to control cancer development in the early stage.

Among cancers, breast cancer is the most frequently diagnosed cancer and the leading cause of cancer death among females in the United States [9]. During tumorigenesis, macrophages, neutrophils, mast cells, myeloid-derived suppressor cells, dendritic cells (DCs), natural killer (NK) cells, and lymphocytes may be recruited due to recurrent inflammation in the tumor microenvironment [10]. NK cells and cytotoxic T cells may destroy tumor cell itself if they can detect the variation of tumor cells [11]. Unfortunately, about 5–10% of all cancer cases result from escaping from surveillance by NK and cytotoxic T cells [12]. Immune stimulation by biological response modifiers (BRMs) may enhance the ability of NK or cytotoxic T cells to remove cancer cells *in vivo*. Moreover, a decrease of inflammation in the tumor microenvironment may be another possible target to pharmaceutically reduce tumorigenesis and angiogenesis in the later stage. Recently, new and possible effective anticancer compounds from natural sources with anti-inflammatory potential such as polysaccharides have been introduced in the treatment of human breast cancer cells via immunotherapy of splenocytes [13]. Potent anti-inflammatory polysaccharides have been a promising agent for anticancer diseases due to regulating M1/M2 or inflammation status in the tumor microenvironment via influencing cytokine secretion profiles by macrophages but not inhibiting the intact immune system.

Polysaccharides, are mostly biological macromolecules in selected fruits, vegetables, and herbal plants, were recently screened for their immunomodulatory activities [6, 14–17]. Polysaccharides extracted from mushrooms [18, 19], fungi [20], algae [21] and other plants [22, 23] were proved to have immunomodulatory and anti-inflammatory activities. Most recently, we found five novel polysaccharides through macrophage-conditioned media (MCM) dose-dependently enhance their inhibitory effects on PC-3 cell growth, implying that macrophages suppress PC-3 cells through decreasing secretion ratios of proinflammatory/anti-inflammatory cytokines in a tumor microenvironment [24].

Even though five potential polysaccharides from guava seed (GSPS), common buckwheat (CBPS), bitter buckwheat (BBPS), red Formosa lambsquarters (RFLPS), and yellow Formosa lambsquarters (YFLPS) were found to have immunomodulatory activities [13, 24], their effects on M1/M2 polarization, LPS-induced inflammation, and MCF-7 breast cancer cell growth using immunotherapy remain elusive. To unravel the puzzle, GSPS, BBPS, CBPS, RFLPS, and YFLPS were selected to evaluate their M1/M2 polarization, anti-inflammatory potential, and anti-MCF-7 breast cell growth using primary peritoneal macrophages in the absence or presence of 5 selected polysaccharides. The correlation between MCF-7 cell viabilities and M1/M2 cytokine secretion profiles in the corresponding MCM were analyzed.

## 2. Materials and Methods

### 2.1. Polysaccharides Preparation

Five selected polysaccharides isolated from guava seed (GSPS), common buckwheat (CBPS), bitter buckwheat (BBPS), red Formosa lambsquarters polysaccharides (RFLPS), and yellow Formosa lambsquarters (YFLPS) were prepared as described previously [13, 24] for characterizing their effects on M1/M2 polarization, LPS-induced inflammation, and MCF-7 breast cancer cell growth via immunotherapy. The constituents and molecular weights of five selected polysaccharides used in this study have been partially characterized [13].

### 2.2. Isolation of Mouse Primary Peritoneal Macrophages

Mouse primary peritoneal macrophages were isolated and prepared as described previously [5]. The peritoneal macrophages from each animal were adjusted to 2 × 10^6^ cells/ml in the tissue culture medium (TCM, a serum replacement; Protide Pharmaceuticals, 1002, Lake Zurich, IL, USA), suspended in a medium containing 20 ml TCM, 1000 ml RPMI 1640 medium (Hyclone, SH30037, South Logan, UT, USA), and 5.0 ml penicillin-streptomycin-amphotericin solution (PSA 100x, Biological Industries, 03-033-1B, Kibbutz Beit Haemek, Israel) consisting of 10,000 U/ml penicillin, 10 mg/ml streptomycin, and 25 *μ*g/ml amphotericin B in 0.85% saline (Atlanta Biologicals Inc.). The cells were counted with a hemocytometer using the trypan blue dye exclusion method. Isolated mouse primary peritoneal macrophages were used for subsequent experiments.

### 2.3. Determination of Noncytotoxic Optimal Concentrations of Five Selected Polysaccharides to Mouse Primary Macrophages Using Cell Viability (MTT Assay)

To determine the noncytotoxic optimal concentrations for the five selected polysaccharide fractions, the cell viabilities of macrophages treated with individual polysaccharides at different concentrations were determined using 3-(4,5-dimethylthiazol-2-diphenyl)-2,5-tetrazolium bromide (MTT, Sigma, MO, USA) assay. All polysaccharide stock solutions were aseptically diluted into working solutions using TCM medium before use. The macrophages (50 *μ*l/well) in the absence or presence of polysaccharide samples (50 *μ*l/well) at the indicated final concentrations of 0, 1.6, 8, 40, 200, 500, and 1000 *μ*g/ml were cultured in 96-well plates and incubated at 37°C in a humidified incubator with 5% CO_2_ and 95% air for 48 h. Lipopolysaccharide (LPS) (Sigma-Aldrich Co., L2654, St. Louis, MO, USA), an endotoxin, was selected at the indicated concentration of 2.5 *μ*g/ml as a positive control in each experiment. LPS is a well-known immunostimulatory agent; therefore, LPS was selected as a positive control for viable macrophages [25]. After incubation, aliquots of 10 *μ*l of MTT (5 mg/ml in phosphate-buffered saline (PBS, 137 mM NaCl, 2.7 mM KCl, 8.1 mM Na_2_HPO_4_, 1.5 mM KH_2_PO_4_, pH 7.4, 0.22 *μ*m filtered) were added to each well in the 96-well plate. The plates were incubated at 37°C in a humidified incubator with 5% CO_2_ and 95% air for another 4 h. After incubation, the plates were centrifuged at 400 × *g* for 10 min. The culture medium was then discarded and carefully washed with PBS buffer twice. Aliquots of 100 *μ*l dimethyl sulfoxide (DMSO) were added to each well and oscillated for 30 min to extract formed insoluble formazan. The absorbance (*A*) was measured at 550 nm on a plate reader (ELISA reader, ASYS Hitech, GmbH, Austria). The cell viability was expressed as the relative percentage (%) compared to the mean absorbency of the control. The cell viability (%) in each biological determination was calculated using the equation: cell viability (% of control) = ((A_sample_ − A_blank_)/(A_control_ − A_blank_)) × 100. The noncytotoxic optimal doses of individual polysaccharides were selected to conduct cytokine secretion assessments. Based on the results of MTT assay, noncytotoxic optimal administration concentrations for the five selected polysaccharides were determined.

### 2.4. Effects of Five Selected Polysaccharides at Their Optimal Administration Concentrations on M1 (Pro-) and M2 (Anti-Inflammatory) Cytokine Secretions by Macrophages in the Absence or Presence of LPS

GSPS, CBPS, BBPS, RFLPS, and YFLPS polysaccharides at their optimal administration concentrations were cultured with peritoneal macrophages in the absence or presence of LPS to assess possible M1/M2 polarization and anti-inflammatory effects using the following two experimental models:

  Model A: mouse peritoneal macrophages (0.5 ml/well) were cocultured with GSPS, CBPS, BBPS, RFLPS, and YFLPS (0.5 ml/well) at their noncytotoxic optimal administration concentrations in 24-well plates and incubated at 37°C in a humidified incubator with 5% CO_2_ and 95% air for 48 h. Endotoxin LPS (L-2654, Sigma-Aldrich Co., St. Louis, MO, USA) was selected as a positive control at 2.5 *μ*g/ml in the culture in each experiment. The supernatants in the cell cultures were collected and stored at −80°C for following M1/M2 cytokine assays.
  Model B: an inflammation-concurrent cell culture model was designed using LPS addition to the test samples. An endotoxin LPS was selected to stimulate inflammation in macrophages. The peritoneal macrophages were cultured in the presence of LPS at 2.5 *μ*g/ml and polysaccharide samples at their noncytotoxic optimal administration concentrations. The plates were incubated in a humidified incubator with 5% CO_2_ and 95% air at 37°C for 48 h. The supernatants in the cell cultures were collected and stored at −80°C for following pro- and anti-inflammatory cytokine assays.

#### 2.4.1. Assays of Pro- and Anti-Inflammatory Cytokines Using ELISA

M1 (proinflammatory) (IL-1*β*, IL-6, and TNF-*α*) concentrations and M2 (anti-inflammatory) (IL-10) cytokines secreted by the mouse peritoneal macrophages were measured using Sandwich ELISA kits (mouse DuoSet ELISA Development system, R&D Systems, Minnesota, USA).

### 2.5. Preparation of Macrophage-Conditioned Media (MCM) in the Absence or Presence of Five Selected Polysaccharides

To prepare MCM, isolated peritoneal macrophages (2 × 10^6^ cells/ml TCM medium, 0.5 ml/well) were cocultured with GSPS, CBPS, BBPS, RFLPS, or YFLPS at the indicated noncytotoxic concentrations of 0, 8, 40, and 200 *μ*g/ml TCM medium (0.5 ml/well) in 24 well plates [24]. The plates were incubated at 37°C in a humidified incubator with 5% CO_2_ and 95% air for 48 h. The cultured plate was centrifuged at 400 ×*g* for 10 min to collect the supernatant (ca. 1.0 ml/well) in the cell cultures, which comprised the macrophage-conditioned medium (MCM). The supernatant of cell cultures was collected and lyophilized. The lyophilized MCM was dissolved in 0.5 ml MEM/EBSS medium. The two-fold concentrated MCM was stored at −80°C until use.

### 2.6. Culture of Human Breast Cancer MCF-7 Cells

Human breast cancer MCF-7 cells were obtained from Bioresource Collection and Research Center (BCRC) in the Food Industry Research and Development Institute (FIRDI), Hsinchu, Taiwan, ROC. The MCF-7 cells were quickly defrosted at 37°C and maintained in the medium of MEM/Earle's balanced salts solution (MEM/EBSS) supplemented with 10% fetal bovine serum (FBS), 0.1 mM nonessential amino acid, 1.0 mM sodium pyruvate, penicillin 100 units/ml, streptomycin 100 *μ*g/ml, and amphotericin B 0.25 *μ*g/ml, at 37°C in a humidified incubator with 95% air and 5% CO_2_. After the cells had grown to 90% confluence in a 75 T tissue culture flask (TPP Biochrom AG, Trasadingen, Switzerland), they were plated at a density of 2 × 10^5^ cells/ml in the 96-well plates to perform the following bio-assay.

### 2.7. Effect of MCM Using Five Different Polysaccharides on the Cell Viability of MCF-7 Cells

To evaluate effects of MCM administration on the cell viability of MCF-7 cells, MCF-7 cells (50 *μ*l/well) were treated with MCM (50 *μ*l/well) or paclitaxel at 2.5 *μ*Μ as a positive control. The plates were incubated in a humidified incubator with 5% CO_2_ and 95% air at 37°C for 24 or 48 h. The live cells were measured by MTT assay. The cell viability (%) in each biological determination was calculated using the equation: cell viability (% of control) = ((A_sample_ − A_blank_)/(A_control_ − A_blank_)) × 100 [26].

### 2.8. Statistical Analysis

Results are expressed as the mean ± standard deviation (SD). Differences among treatments were analyzed using one-way analysis of variance (ANOVA), followed by Duncan's multiple range test using the SPSS system with 19.0. The relationship between cytokine levels in MCM and cell viabilities of MCF-7 cells was described as Pearson product-moment correlation coefficient (*r*). *P* < 0.05 considered a significant difference.

## 3. Results and Discussion

### 3.1. Noncytotoxic Optimal Concentrations of Five Selected Polysaccharides for Primary Peritoneal Macrophages

**Figure 1:**
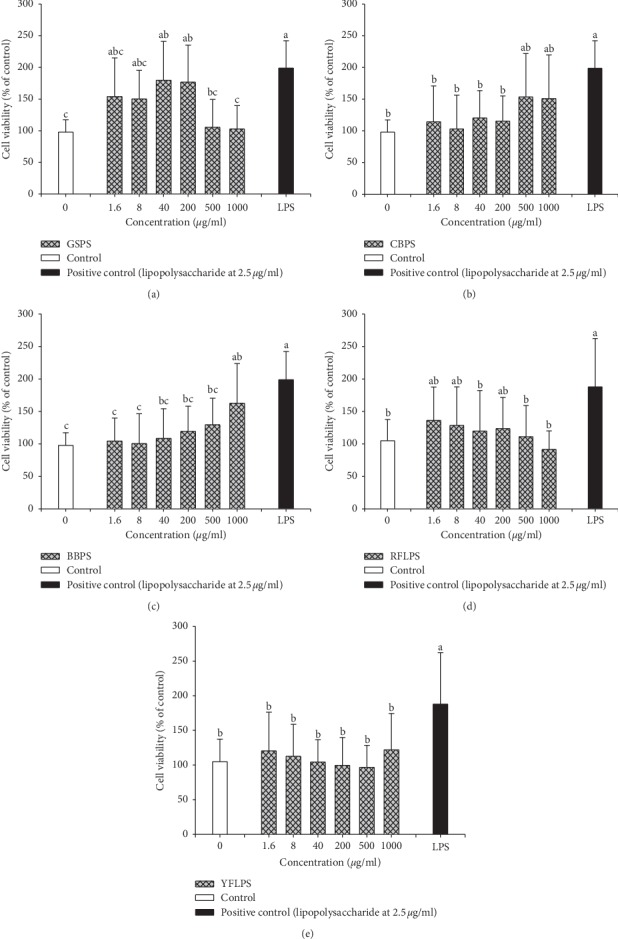
Cell viabilities of macrophages treated with five selected polysaccharides (a) GSPS, (b) CBPS, (c) BBPS, (d) RFLPS, and (e) YFLPS. Values are means ± SD (*n* = 6 biological determinations). Bars not sharing a common letter are significantly different (*P* < 0.05) from each other analyzed by one-way ANOVA, followed by Duncan's multiple range test. Each cell population (1 × 10^6^ cells/ml) was, respectively, treated with the polysaccharides at the indicated concentrations for 48 h The lipopolysaccharide (LPS, 2.5 *μ*g/ml) was selected as a positive control. The cell viability was determined using MTT assay and calculated using the equation: cell viability (% of control) = ((A_sample_ − A_blank_)/(A_control_ − A_blank_)) × 100.

To avoid cytotoxicity from excess concentration administration for the five selected polysaccharides, GSPS, CBPS, BBPS, RFLPS, and YFLPS at indicated concentrations of 0, 1.6, 8, 40, 200, 500, and 1000 *μ*g/ml, were cocultured with primary peritoneal macrophages for 48 h, respectively. The LPS at 2.5 *μ*g/ml was selected as a positive control. The cell viability was assayed using MTT assay. The results showed that all GSPS, CBPS, BBPS, RFLPS, and YFLPS administrations at the indicated concentrations did not significantly (*P* > 0.05) exhibit any cytotoxicity to macrophages compared to that of the control, respectively (Figure 1). Interestingly, GSPS and BBPS treated at appropriate concentrations significantly (*P* < 0.05) increased the macrophage cell viability, suggesting that some of these selected polysaccharides have immunostimulatory potential (Figures 1(a) and 1(c)). In contrast, GSPS and RFLPS treated at the indicated concentrations of 500 and 1000 *μ*g/ml slightly decreased (*P* > 0.05) the macrophage cell viability (Figures 1(a) and 1(d)). To avoid unpredictable cytotoxicities at the higher doses, GSPS, CBPS, BBPS, RFLPS, and YFLPS at the same concentrations of 1.6, 8, 40, and 200 *μ*g/ml were selected as optimal concentrations for treating macrophages and used for the following cytokine secretion assays and MCM preparation.

### 3.2. Effects of Five Selected Polysaccharides on Cytokine Secretion Profiles by Primary Peritoneal Macrophages in the Absence or Presence of LPS

**Figure 2:**
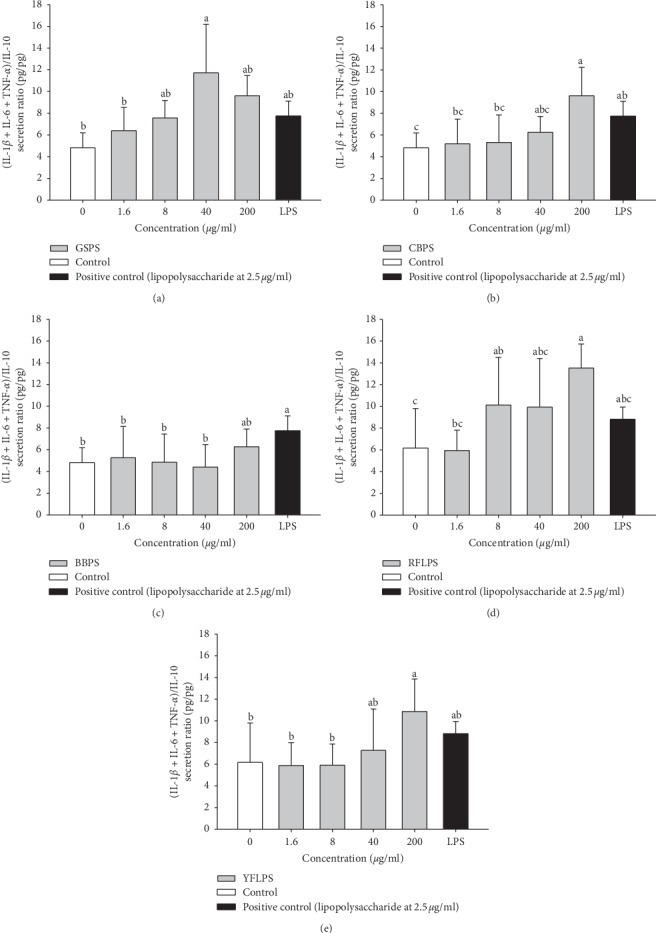
Effect of treatments with five selected polysaccharides (a) GSPS, (b) CBPS, (c) BBPS, (d) RFLPS, and (e) YFLPS on M1/M2 cytokine secretion ratios by peritoneal macrophages from female BALB/c mice. Values are means ± SD (*n* = 8 biological determinations). Bars without sharing a common letter are significantly different (*P* < 0.05) from each other analyzed by one-way ANOVA, followed by Duncan's multiple range test. Each cell population (1 × 10^6^ cells/ml medium) was, respectively, treated with the polysaccharides at the indicated concentrations for 48 h. Lipopolysaccharide (LPS, 2.5 *μ*g/ml) treatment alone was selected as a positive control.

To evaluate possible M1/M2 polarization and anti-inflammatory potential of the five selected polysaccharides, GSPS, CBPS, BBPS, RFLPS, and YFLPS at the indicated noncytotoxic optimal concentrations of 1.6, 8, 40, and 200 *μ*g/ml were selected to treat macrophages for 48 h in the absence or presence of LPS. Cytokine secretion profiles including M1 (pro-) (IL-1*β*, IL-6, and TNF-*α*) and M2 (anti-inflammatory) (IL-10) cytokines from primary peritoneal macrophages were measured, respectively.  Table 1 shows the effect of five different polysaccharides on M1 and M2 cytokine secretions by peritoneal macrophages from female BALB/c mice. LPS treatment (2.5 *μ*g/ml) alone was selected as a positive control. The results showed that IL-1*β*, IL-6, TNF-*α*, and IL-10 secretions by the LPS-stimulated macrophages significantly (*P* < 0.05) increased as compared to those of negative controls, indicating that the cell culture model used in this study is suitable and effective for M1/M2 bioassay (Table 1). Interestingly, both M1 and M2 cytokine secretion levels by the primary peritoneal macrophages treated with five selected polysaccharides dose-dependently and significantly (*P* < 0.05) increased, suggesting that all five selected polysaccharides have immunostimulatory potential, particularly GSPS, RFLPS, and CBPS in order (Table 1). In traditional Chinese herbal medicine, some active ingredients are proven polysaccharides [6]. Parts of the polysaccharides are considered BRMs for enhancing immune activity [27]. In the present study, we evidence that GSPS, CBPS, and RFLPS among the five selected polysaccharides presented substantial immunostimulatory effects on macrophages. Our study suggests that GSPS, CBPS, and RFLPS showed substantial immunostimulatory effects on macrophages that may be applicable to BRMs in the future. Further analysis on M1/M2 cytokine secretion ratios showed that RFLPS, GSPS, YFLPS, and CBPS in order significantly (*P* < 0.05) increased M1 (IL-1*β* + IL-6 + TNF-*α*)/M2 (IL-10) secretion ratios as compared to that of the control, suggesting that these four selected polysaccharides treated alone might result in macrophage activation and reveal a M1 polarization property (Figure 2).

**Figure 3:**
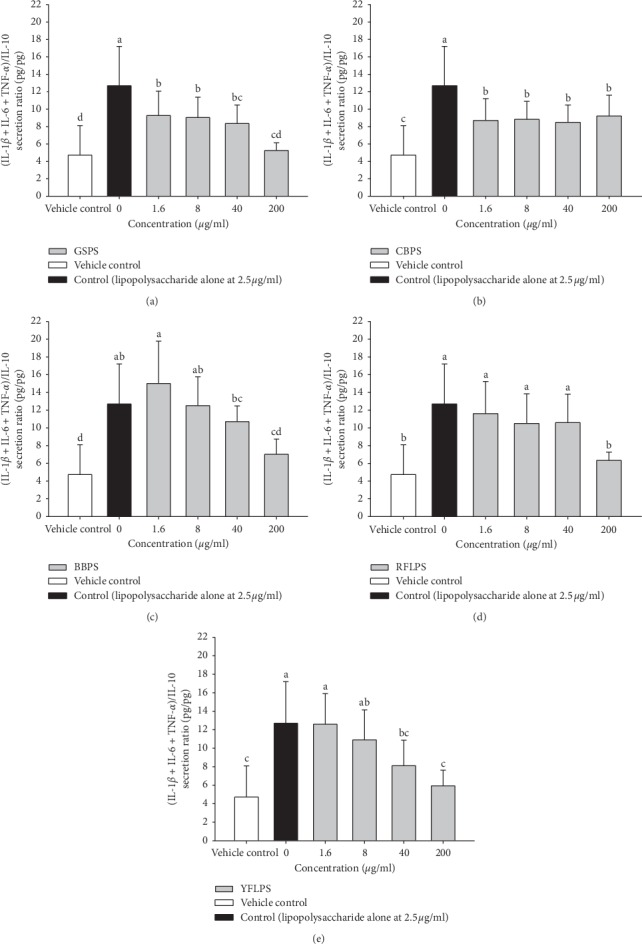
Effect of treatments with five selected polysaccharides (a) GSPS, (b) CBPS, (c) BBPS, (d) RFLPS, and (e) YFLPS on pro-/anti-inflammatory cytokine secretion ratios by LPS-stimulated peritoneal macrophages from female BALB/c mice. Values are means ± SD (*n* = 8 biological determinations). Bars without sharing a common letter are significantly different (*P* < 0.05) from each other analyzed by one-way ANOVA, followed by Duncan's multiple range test. Each cell population (1 × 10^6^ cells/ml medium) was, respectively, treated with the polysaccharides at the indicated concentrations in the presence of lipopolysaccharide (2.5 *μ*g/ml) for 48 h vehicle control, VC (cells alone).

To examine anti-inflammatory potential of the five selected polysaccharides against LPS-stimulated inflammation, GSPS, CBPS, BBPS, RFLPS, and YFLPS polysaccharides at the indicated noncytotoxic optimal concentrations were administered to mouse primary peritoneal macrophages in the presence of LPS for 48 h. Importantly, all five selected polysaccharides significantly (*P* < 0.05) increased IL-10 (an anti-inflammatory cytokine) levels in cultures of LPS-stimulated macrophages, suggesting that these selected polysaccharides have anti-inflammatory potential. Unfortunately, IL-6 levels were also significantly (*P* < 0.05) increased by GSPS and BBPS. Most importantly, GSPS administration significantly (*P* < 0.05) decreased TNF-*α* and IL-6 (proinflammatory cytokines) secretion levels by LPS-stimulated macrophages, suggesting that GSPS might have the most anti-inflammatory potential. Interestingly, GSPS and RFLPS administrations also significantly (*P* < 0.05) increased IL-1*β* (a proinflammatory cytokine) levels in the LPS-stimulated macrophages (Table 2). To avoid confusion, pro- (IL-1*β* + IL-6 + TNF-*α*)/anti-inflammatory (IL-10) cytokine secretion levels by macrophages were further analyzed (Figure 3). The (IL-1*β* + IL-6 + TNF-*α*)/IL-10 cytokine secretion ratio in the LPS-stimulated macrophages significantly (*P* < 0.05) increased as compared to that of the vehicle control (VC). All five selected polysaccharides significantly (*P* < 0.05) decreased the (IL-1*β* + IL-6 + TNF-*α*)/IL-10 cytokine secretion ratio in the LPS-stimulated macrophages, suggesting that these selected polysaccharides have anti-inflammatory potential (Figure 3). According to decreases in the (IL-1*β* + IL-6 + TNF-*α*)/IL-10 cytokine secretion ratios at 200 *μ*g/ml of polysaccharides, GSPS showed the most anti-inflammatory potential among the five selected polysaccharides. The polysaccharide purified from *Ecklonia cava* extract was shown to dose-dependently inhibit LPS-induced inducible nitric oxide synthase (iNOS) and cyclooxygenase-2 (COX-2) gene expression, as well as the subsequent production of NO and prostaglandin E2 (PGE2) by LPS in RAW 264.7 macrophages [28]. Our results exhibited that all five selected polysaccharide fractions, particularly GSPS, had anti-inflammatory potential (Figure 3), via decreasing the (IL-1*β* + IL-6 + TNF-*α*)/IL-10 cytokine secretion ratio in LPS-stimulated macrophages. GSPS exhibited the greatest immuno-stimulatory and anti-inflammatory effects among the five selected polysaccharides (Figures 2 and 3).

### 3.3. Effects of MCM Prepared in the Absence or Presence of Five Selected Polysaccharides on MCF-7 Cell Growth

**Figure 4:**
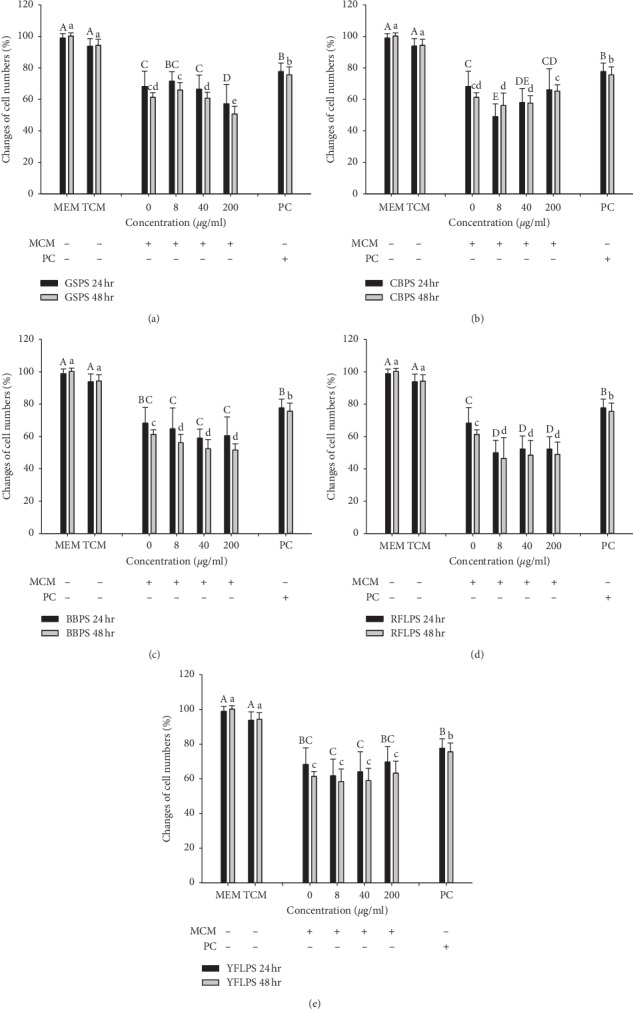
Effect of MCM prepared in the absence or presence of five selected polysaccharides (a) GSPS, (b) CBPS, (c) BBPS, (d) RFLPS, and (e) YFLPS on MCF-7 cell growth. MCF-7 cells (2 × 10^5^ cells/ml) were treated with MCM for 24 or 48 h respectively. Values are mean ± SD (*n* = 6 biological determinations). Bars at the same incubation time not sharing a common letter are significantly different (*P* < 0.05) from each other assayed by one-way ANOVA, followed by Duncan's multiple range test. MCM, macrophage-conditioned medium. PC, positive control (paclitaxel at 2.5 *μ*Μ).

We found that direct administrations of the five selected polysaccharides could not markedly influence the growth of MCF-7 cells [13]. To further evaluate possible effects of the five selected polysaccharides on breast cancer via tumor immunotherapy, MCF-7 cells were treated with MCM prepared with the five selected polysaccharides at the indicated noncytotoxic concentrations of 0, 8, 40, and 200 *μ*g/ml (Figure 4). The results showed that there was no significant difference in the viabilities of MCF-7 cells between cultured with MEM/EBSS (medium for the cancer cell line) and TCM medium (medium for mouse primary macrophages), indicating that TCM medium in the MCM did not influence MCF-7 cell growth. Paclitaxel (a treatment control) significantly (*P* < 0.05) inhibited MCF-7 cell growth as compared to that of negative control (cells alone either in MEM/EBSS or TCM medium) through 24 or 48 h incubation, implying that paclitaxel might be applied for breast cancer treatment. Importantly, the control group (MCM without polysaccharides) significantly (*P* < 0.05) inhibited the MCF-7 cell viability as compared to that of negative control through 24 or 48 h incubation, implying that secretions by macrophages, e.g., cytokines, might influence MCF-7 cell growth. Individually, MCM prepared with GSPS at 40 and 200 *μ*g/ml significantly (*P* < 0.05) and dose-dependently enhanced their inhibitory effects on MCF-7 cell viabilities through 48 h incubation (Figure 4(a)). Incubation with MCM prepared with CBPS at 8 *μ*g/ml for 24 h significantly (*P* < 0.05) enhanced its inhibitory effect on MCF-7 cell viabilities (Figure 4(b)). MCM prepared with BBPS at the indicated concentrations for 48 h significantly (*P* < 0.05) enhanced their inhibitory effects on MCF-7 cell viabilities (Figure 4(c)). MCM prepared with RFLPS at the indicated concentrations either through 24 or 48 h incubation significantly (*P* < 0.05) enhanced its inhibitory effects on MCF-7 cell viability (Figure 4(d)). However, MCM prepared with YFLPS at the indicated concentrations could not enhance its inhibitory effects on MCF-7 cell viability either through 24 or 48 h incubation (Figure 4(e)). Among five selected polysaccharides, GSPS had the highest potential for anti-breast cancer via cancer immunotherapy by modulating macrophages secretion.

Recently, certain plant polysaccharides have been found to enhance or activate the immune response of macrophages. DDP1-1 obtained from *Dendrobium denneanum* polysaccharides enhances a curative effect on cancer and may be served as a novel anticancer drug [29]. SCP-60, a water-extracted crude polysaccharide from *Sarcodia ceylonensis*, exhibits a strong antitumor activity in S180 mice [30]. In the present study, our results suggest that MCM cultured with selected polysaccharides (except YFLPS) markedly enhanced their inhibitory effects on MCF-7 cell growth, indicating that these four polysaccharides, particularly GSPS, may be served as a novel potential anti-breast-cancer drug via tumor immunotherapy.

### 3.4. Association between MCF-7 Cell Viabilities and Cytokine Levels in the Corresponding MCM

**Figure 5:**
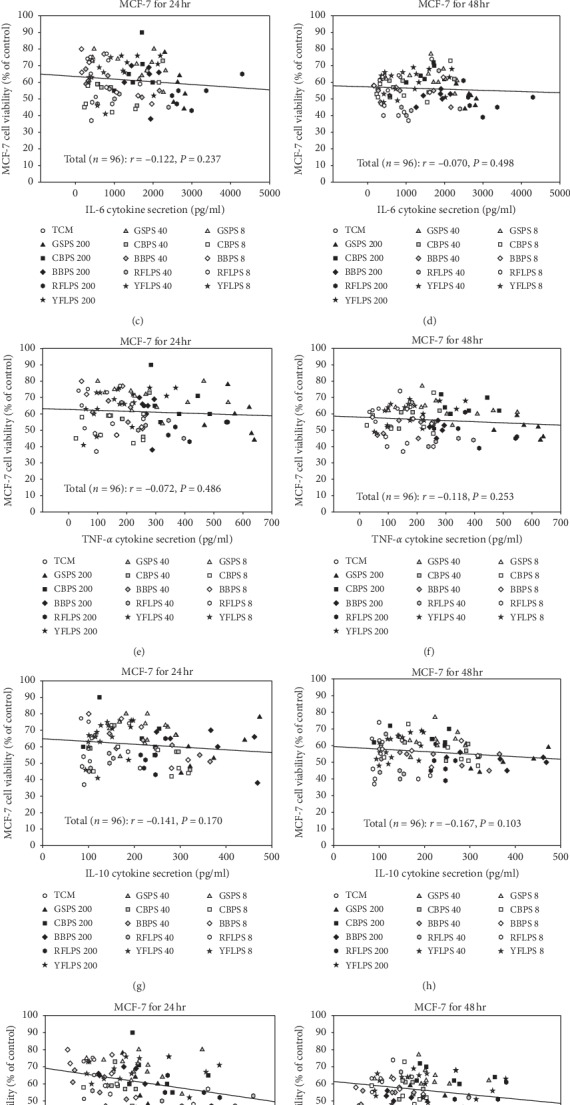
The association between breast cancer MCF-7 cell viability and (a, b) IL-1*β*, (c, d) IL-6, (e, f) TNF-*α*, (g, h) IL-10 levels and (i, j) (IL-6 + TNF-*α*)/IL-10 cytokine secretion ratios in the corresponding MCM incubated for 24 or 48 h ^*∗*^*P* < 0.05; ^*∗∗*^*P* < 0.001. IL, interleukin; MCM, macrophage-conditioned media cultured with 5 selected polysaccharides at the indicated concentrations of 0, 8, 40, and 200 *μ*g/ml, respectively. The symbols in the graphs represent that the 5 selected polysaccharides at different concentrations were added to each individual MCM, respectively.

We presumed that cytokine secretions by macrophages in MCM might influence MCF-7 cell growth. To clarify association between cytokine levels in the MCM and MCF-7 cell viabilities, the correlations between the cytokine levels in their corresponding MCM and the survival rate of the breast cancer cells treated with MCM were determined using Pearson's correlation coefficients (*r*) (Figure 5). The results showed that there were no significant correlations between secretion levels of individual proinflammatory cytokines IL-1*β*, IL-6, and TNF-*α* (M1-type cytokines), as well as an anti-inflammatory cytokine IL-10 (M2-type cytokine) in the MCM and their corresponding MCF-7 cell viabilities through 24 or 48 h incubation (Figures 5(a)–5(h)). Most importantly, there was a significantly negative correlation between M1 (IL-6 + TNF-*α*)/M2 (IL-10) cytokine secretion ratios and MCF-7 cell viabilities either through 24 h (*r* = −0.307, *P*=0.002) (Figure 5(i)) or 48 h incubation (*r* = −0.263, *P*=0.010) (Figure 5(j)). Even though low mutuality, our results evidence that increased M1/M2 cytokine ratios in the tumor microenvironment significantly inhibited the growth of human breast MCF-7 cancer cells. Increased M1/M2 cytokine ratios revealed that M1 macrophages might dominate in the MCM that enhanced inflammation via producing proinflammatory cytokines [7]. Increased inflammation status, namely, M1 macrophages dominate, may help to control cancer development in the early stage. We found that RFLPS, GSPS, YFLPS, and CBPS in order significantly (*P* < 0.05) increased M1/M2 cytokine secretion ratios, suggesting that these four selected polysaccharides treated alone enhanced macrophage activation and inflammation (Figure 2). Consequently, particular polysaccharides including RFLPS, GSPS, YFLPS, and CBPS may increase the cell number of M1 macrophages in the tumor microenvironment and inhibit MCF-7 cell growth via increasing M1/M2 cytokine secretion ratios.

Currently, cancer immunotherapy is becoming a viable alternative to traditional cancer treatment options [31, 32]. Our results exhibited that cytokine immunotherapy, particularly M1-type cytokines, might be effective to inhibit human breast cancers (Figures 4 and 5). Numerous animal tumor model studies have demonstrated that cytokines have broad antitumor activity [33]. Intervention of polysaccharides to immune cells, particularly macrophages, may modulate their cytokine secretion profiles. Polysaccharides from *Cymbopogon citratus* (CCPS) have been found to inhibit the growth of transplanted S180 tumors, improve the immunity of tumor-bearing mice and increase IL-2, IL-6, IL-12, and TNF-*α* cytokine secretions, suggesting that CCPS may have an antitumor activity via modulating cytokine secretion [34]. In addition, traditional oriental medicine such as *Portulaca oleracea* L. has known for its immune-stimulatory effect through upregulating inflammatory cytokines (IL-2, IL-12, TNF-*α*, and IFN-*γ*) and NK cell activity *in vitro* [35]. In the present study, we demonstrated that MCM prepared with four selected polysaccharides including GSPS, CBPS, BBPS, and RFLPS enhanced their inhibitory effects on MCF-7 cell growth, possibly via cytokine immunotherapy. We hypothesized that particular polysaccharides might modulate cytokine secretion profiles of macrophages and subsequently change a microenvironment to inhibit tumor growth. Importantly, RFLPS and GSPS had the highest antitumor potential among the five selected polysaccharides, via regulating macrophages cytokine secretion. Differentiated macrophages (M1 or M2) can be easily discriminated by their unique cytokine secretion profiles [36]. In the present study, we investigated the main functions of polarized macrophages (M1 or M2) in cancer diseases through correlating cytokine secretion profiles and MCF-7 cell growth. Our results provide the evidence to infer that increased M1 in the tumor microenvironment may inhibit breast cancer MCF-7 cell growth. Moreover, the polarization of different macrophages can be stimulated by adding certain stimuli such as polysaccharides.

The five selected polysaccharides have been partially characterized [13]. We presumed that these selected polysaccharides, particularly RFLPS and GSPS, are a mixture of nonstarch galactan, possibly proteopolysaccharides [13]. Macrophages in Peyer's patches may be activated by particular polysaccharides via certain receptors, such as glucan receptors on macrophages. Once immune cells are firstly activated by potent polysaccharides in the gut, indirect prevention or treatment of cancer by the activated immune cells may happen, namely, tumor immunotherapy.

## 4. Conclusions

All of the selected polysaccharides, except BBPS, significantly and dose-dependently increased (IL-1*β* + IL-6 + TNF-*α*)/(IL-10) cytokine secretion ratios by macrophages, suggesting that these four selected polysaccharides have M1 polarization property and activate slight inflammation in macrophages. Treatments using the five selected polysaccharides in the presence of LPS significantly decreased (IL-1*β* + IL-6 + TNF-*α*)/(IL-10) cytokine secretion ratios in macrophages, suggesting that all five selected polysaccharide fractions, particularly GSPS, have anti-inflammatory potential. All MCM prepared with selected polysaccharides (except YFLPS) significantly (*P* < 0.05) enhanced their inhibitory effects on MCF-7 cell growth. A negative correlation was noted between MCF-7 cell viabilities and M1/M2 cytokine secretion ratios ((IL-6 + TNF-*α*)/IL-10) in the corresponding MCM, suggesting that increases in M1 macrophages in the tumor microenvironment might inhibit MCF-7 cell growth. Particular polysaccharides, including RFLPS, GSPS, YFLPS, and CBPS, may increase the percentage of M1 macrophages in the tumor environment to inhibit MCF-7 cell growth.

## Figures and Tables

**Table 1:** Effect of treatments with five different polysaccharides on M1 and M2 cytokine secretions by peritoneal macrophages from female BALB/c mice^a,b,c^.

Cytokine^d^ (pg/ml)	Conc (*μ*g/ml)	GSPS^f^	CBPS^f^	BBPS^f^	RFLPS^f^	YFLPS^f^
IL-1*β*	0	ND	ND	ND	ND	ND
	1.6	ND	4.2 ± 3.6^C^	3.3 ± 2.8^C^	1.41 ± 1.26^E^	ND
	8	ND	5.5 ± 4.0^C^	3.6 ± 3.0^C^	16.7 ± 16.3^D^	1.8 ± 1.6^CD^
	40	57.4 ± 40.8^C^	6.6 ± 5.1^C^	5.4 ± 2.7^C^	34.7 ± 11.3^C^	7.2 ± 3.7^C^
	200	294 ± 44.1^A^	17.5 ± 12.5^B^	14.9 ± 6.7^B^	52.6 ± 7.4^B^	26.6 ± 5.7^B^
	LPS^e^	108 ± 19.3^B^	108 ± 19.3^B^	108 ± 19.3^B^	67.8 ± 12.3^A^	67.8 ± 12.3^A^

IL-6	0	552 ± 217^D^	552 ± 217^D^	552 ± 217^D^	646 ± 346^C^	646 ± 346^C^
	1.6	931 ± 615^D^	1036 ± 750^BC^	1035 ± 795^C^	655 ± 380^C^	671 ± 336^C^
	8	981 ± 516 ^D^	1097 ± 760^BC^	1027 ± 766^C^	1350 ± 975^C^	743 ± 426^C^
	40	1994 ± 209^C^	1202 ± 613^B^	1077 ± 755^C^	1194 ± 649^C^	825 ± 354^C^
	200	2608 ± 185^B^	1573 ± 299^B^	1806 ± 318^B^	3019 ± 659^B^	1768 ± 398^B^
	LPS^e^	5332 ± 644^A^	5332 ± 644^A^	5332 ± 644^A^	5471 ± 1018^A^	5471 ± 1018^A^

TNF-*α*	0	81 ± 49^D^	81 ± 49^D^	81 ± 49^D^	58 ± 39^D^	58 ± 39^D^
	1.6	171 ± 112^C^	176 ± 101^C^	160 ± 113^CD^	98 ± 38^D^	90 ± 31^C^
	8	209 ± 83^C^	182 ± 106^C^	148 ± 93^CD^	196 ± 99^C^	114 ± 43^C^
	40	378 ± 95^B^	216 ± 60^C^	176 ± 84^C^	237 ± 99^C^	141 ± 60^C^
	200	583 ± 60^A^	365 ± 78^B^	266 ± 21^B^	383 ± 112^B^	247 ± 73^B^
	LPS^e^	624 ± 91^A^	624 ± 91^A^	624 ± 91^A^	496 ± 146^A^	496 ± 146^A^

IL-10	0	131 ± 38^C^	131 ± 38^C^	131 ± 38^C^	118 ± 14^C^	118 ± 14^C^
	1.6	166 ± 82^C^	213 ± 87^BC^	200 ± 83^CD^	109 ± 30^C^	131 ± 51^C^
	8	175 ± 75^C^	224 ± 83^B^	215 ± 81^CD^	149 ± 63^C^	120 ± 26^C^
	40	203 ± 82^C^	228 ± 81^B^	264 ± 81^C^	151 ± 48^C^	127 ± 18^C^
	200	315 ± 120^B^	190 ± 62^BC^	365 ± 86^B^	237 ± 20^B^	181 ± 18^B^
	LPS^e^	812 ± 128^A^	812 ± 128^A^	812 ± 128^A^	656 ± 83^A^	656 ± 83^A^

^a^Values are means ± SD (*n* = 8 biological determinations). ^b^Values within same column in the same cytokine item not sharing a common capital superscript letter are significantly different (*P* < 0.05) from each other analyzed by one-way ANOVA, followed by Duncan's multiple range test. ^c^Each cell population (1 × 10^6^ cells/ml medium) was, respectively, treated with the polysaccharides at the indicated concentrations for 48 h. ^d^The limit of detection of cytokine ELISA kits used in this study was <15.6 pg/ml. ^e^The lipopolysaccharide (LPS at 2.5 *μ*g/ml) was selected as a positive control. ^f^GSPS, guava seed polysaccharides; CBPS, common buckwheat polysaccharides; BBPS, bitter buckwheat polysaccharides; RFLPS, red Formosa lambsquarters polysaccharides; YFLPS, yellow Formosa lambsquarters polysaccharides; ND, not detectable.

**Table 2:** Effect of treatments with five different polysaccharides on pro- and anti-inflammatory cytokine secretions by lipopolysaccharide-stimulated peritoneal macrophages from female BALB/c mice^a,b,c^.

Cytokine^d^ (pg/ml)	Conc (*μ*g/ml)	GSPS^f^	CBPS^f^	BBPS^f^	RFLPS^f^	YFLPS^f^
IL-1*β*	VC^e^	2.85 ± 2.44^D^	2.85 ± 2.44^B^	2.85 ± 2.44^B^	2.85 ± 2.44^D^	2.85 ± 2.44^B^
	0	58 ± 12.3^C^	58 ± 12.3^A^	58 ± 12.3^A^	58 ± 12.3^C^	58 ± 12.3^A^
	1.6	54.5 ± 10.7^C^	63.9 ± 11.8^A^	59.8 ± 11.0^A^	64.0 ± 8.8^BC^	60.9 ± 17.1^A^
	8	57.3 ± 12.8^C^	61.0 ± 11.0^A^	54.9 ± 11.1^A^	64.6 ± 19.2^BC^	65.5 ± 173^A^
	40	135 ± 31.4^B^	61.5 ± 13.2^A^	53.6 ± 10.9^A^	91.8 ± 18.5^A^	77.6 ± 22.7^A^
	200	293 ± 66.8^A^	64.0 ± 12.5^A^	61.8 ± 11.6^A^	80.1 ± 22.4^AB^	75.9 ± 22.7^A^

IL-6	VC^e^	441 ± 261^C^	441 ± 261^B^	441 ± 261^C^	441 ± 261^B^	441 ± 261^B^
	0	5496 ± 1027^A^	5496 ± 1027^A^	5496 ± 1027^B^	5496 ± 1027^A^	5496 ± 1027^A^
	1.6	5094 ± 1137^AB^	4858 ± 1104^A^	7531 ± 1372^A^	5645 ± 1473^A^	6784 ± 1175^A^
	8	4947 ± 982^AB^	5304 ± 1177^A^	7032 ± 1635^A^	5834 ± 1615^A^	6058 ± 1326^A^
	40	4724 ± 999^AB^	5632 ± 1578^A^	7769 ± 665^A^	6762 ± 1500^A^	5738 ± 1658^A^
	200	4236 ± 713^B^	6142 ± 703^A^	8402 ± 1456^A^	5836 ± 993^A^	5692 ± 1465^A^

TNF-*α*	VC^e^	51 ± 20^C^	51 ± 20^C^	51 ± 20^B^	51 ± 20^B^	51 ± 20^B^
	0	577 ± 133^A^	577 ± 133^AB^	577 ± 133^A^	577 ± 133^A^	577 ± 133^A^
	1.6	569 ± 158^AB^	669 ± 200^A^	481 ± 243^A^	808 ± 427^A^	739 ± 337^A^
	8	488 ± 118^AB^	557 ± 154^AB^	433 ± 194^A^	721 ± 353^A^	589 ± 185^A^
	40	495 ± 111^AB^	380 ± 282^B^	402 ± 170^A^	603 ± 171^A^	568 ± 239^A^
	200	429 ± 132^B^	460 ± 161^B^	511 ± 189^A^	589 ± 321^A^	702 ± 397^A^

IL-10	VC^e^	171 ± 104^C^	171 ± 104^C^	171 ± 104^D^	171 ± 104^D^	171 ± 104^D^
	0	536 ± 160^B^	536 ± 160^B^	536 ± 160^C^	536 ± 160^C^	536 ± 160^C^
	1.6	645 ± 193^B^	662 ± 154^AB^	573 ± 143^C^	599 ± 112^BC^	631 ± 164^BC^
	8	630 ± 174^B^	685 ± 163^AB^	611 ± 137^C^	656 ± 172^BC^	643 ± 168^C^
	40	675 ± 218^B^	704 ± 156^AB^	799 ± 128^B^	728 ± 188^B^	823 ± 195^B^
	200	890 ± 232^A^	753 ± 179^A^	1321 ± 174^A^	1041 ± 211^A^	1127 ± 279^A^

^a^Values are means ± SD (*n* = 8 biological determinations). ^b^Values within same column in the same cytokine item not sharing a common capital superscript letter are significantly different (*P* < 0.05) from each other assayed by one-way ANOVA, followed by Duncan's multiple range test. ^c^Each cell population (1 × 10^6^ cells/ml medium) was, respectively, treated with the polysaccharides at the indicated concentrations in the presence of lipopolysaccharide (2.5 *μ*g/ml) for 48 h. ^d^The limit of detection of cytokine ELISA kits used in this study was <15.6 pg/ml. ^e^VC, vehicle control (cells alone). ^f^GSPS, guava seed polysaccharides; CBPS, common buckwheat polysaccharides; BBPS, bitter buckwheat polysaccharides; RFLPS, red Formosa lambsquarters polysaccharides; YFLPS, yellow Formosa lambsquarters polysaccharides.

## Data Availability

The data used to support the findings of this study available from the corresponding author upon request.
